# Zinc Alginate Hydrogel-Coated Wound Dressings: Fabrication, Characterization, and Evaluation of Anti-Infective and In Vivo Performance

**DOI:** 10.3390/gels11060427

**Published:** 2025-06-01

**Authors:** Adelina-Gabriela Niculescu, Alexandra Cătălina Bîrcă, George Dan Mogoşanu, Marius Rădulescu, Alina Maria Holban, Daniela Manuc, Adina Alberts, Alexandru Mihai Grumezescu, Laurenţiu Mogoantă

**Affiliations:** 1 Department of Science and Engineering of Oxide Materials and Nanomaterials, National University of Science and Technology Politehnica Bucharest, 011061 Bucharest, Romania; adelina.niculescu@upb.ro (A.-G.N.); ada_birca@yahoo.com (A.C.B.); marius.radulescu@upb.ro (M.R.); agrumezescu@upb.ro (A.M.G.); 2 Research Institute of the University of Bucharest—ICUB, University of Bucharest, 050657 Bucharest, Romania; 3 Department of Pharmacognosy and Phytotherapy, Faculty of Pharmacy, University of Medicine and Pharmacy of Craiova, 2 Petru Rareş Street, 200349 Craiova, Romania; mogosanu2006@yahoo.com; 4 Drug Research Center, Faculty of Pharmacy, University of Medicine and Pharmacy of Craiova, 2 Petru Rareş Street, 200349 Craiova, Romania; 5 Department of Microbiology, Faculty of Biology, University of Bucharest, 91–95 Independenţei Avenue, 050095 Bucharest, Romania; alina_m_h@yahoo.com; 6 Carol Davila University of Medicine and Pharmacy, 050474 Bucharest, Romania; daniela.manuc@umfcd.ro; 7 Research Center for Microscopic Morphology and Immunology, University of Medicine and Pharmacy of Craiova, 2 Petru Rareş Street, 200349 Craiova, Romania; laurentiu_mogoanta@yahoo.com; 8 Department of Histology, Faculty of Medicine, University of Medicine and Pharmacy of Craiova, 2 Petru Rareş Street, 200349 Craiova, Romania

**Keywords:** zinc alginate, wound healing, bioactive wound dressings, antimicrobial properties, burn wound treatment, fibroblast proliferation

## Abstract

The delayed healing and infection risks associated with chronic wounds and burns pose significant clinical challenges. Traditional dressings provide basic coverage but lack the bioactive properties needed for tissue regeneration and antimicrobial protection. In this study, we developed zinc alginate hydrogel-coated traditional wound dressings (WD@AlgZn) and evaluated their physicochemical properties, antimicrobial performance, and in vivo healing efficacy. Scanning electron microscopy (SEM) revealed a uniform coating of the zinc alginate network on dressing fibers, while Fourier-transform infrared spectroscopy (FT-IR) confirmed the successful incorporation of zinc ions. Antimicrobial assays further demonstrated that WD@AlgZn reduced bacterial loads (CFU/mL counts) by several orders of magnitude for both *Staphylococcus aureus* and *Escherichia coli* compared to uncoated controls. An in vivo rat burn wound model exhibited accelerated wound closure when using WD@AlgZn dressings compared to conventional wound care approaches, achieving a 90.75% healing rate by day 21, significantly outperforming the silver sulfadiazine (52.32%), uncoated-dressing (46.58%), and spontaneous-healing (37.25%) groups. Histological analysis confirmed enhanced re-epithelialization, neovascularization, and reduced inflammation in WD@AlgZn-treated tissues. The findings suggest that WD@AlgZn offers a promising alternative for advanced wound management, combining structural robustness with bioactive properties to support efficient wound healing and infection control. These results provide valuable insights into the potential clinical applications of metal-ion cross-linked biopolymeric hydrogel dressings for next-generation wound care strategies.

## 1. Introduction

Wound healing is a complex biological process that involves the careful coordination of inflammation, tissue regeneration, and the remodeling of the extracellular matrix [1,2]. Chronic wounds, including diabetic ulcers, pressure sores, and severe burns, present significant clinical challenges due to prolonged healing times, increased susceptibility to infections, and impaired vascularization [1,3,4,5,6,7]. The growing prevalence of non-healing wounds has led to an urgent demand for advanced wound care solutions beyond conventional approaches [8,9]. Traditional wound dressings, such as gauze and hydrocolloid-based materials, mainly serve as protective barriers but often fail to actively promote wound healing or prevent microbial contamination [10,11,12]. Maintaining a moist wound environment is necessary for accelerating re-epithelialization and minimizing scarring, while preventing bacterial infections is important, as biofilm formation can significantly hinder wound closure and lead to severe complications [13]. To address these limitations, modern wound dressings must integrate bioactive properties that provide a physical barrier and contribute to antimicrobial defense, cellular stimulation, and tissue regeneration [10,14,15,16,17].

Sodium alginate, a naturally occurring polysaccharide derived from brown seaweed, has gained significant attention for its use in wound dressings due to its excellent biocompatibility, biodegradability, and gel-forming ability [18]. Its unique capacity for ionic cross-linking with divalent cations such as Zn^2+^ enables the formation of stable hydrogel matrices that enhance moisture retention, facilitate cell migration, and provide an optimal healing environment [19,20]. Alginate-based wound dressings are known for absorbing exudates, conforming to wound beds, and supporting tissue regeneration while minimizing trauma during dressing changes [21]. In addition to these mechanical and physical benefits, alginate hydrogels are effective delivery systems for bioactive agents, enabling the controlled release of therapeutic compounds, including antimicrobial metals and wound-healing promoters [22]. Cross-linking alginate with Zn^2+^ improves the hydrogel’s structural stability and biological properties, making it a promising material for advanced wound care applications [23].

Zinc is a trace element in wound healing that regulates collagen synthesis, immune responses, angiogenesis, and epithelialization [24,25]. It is particularly important for fibroblast activity, keratinocyte migration, and extracellular matrix remodeling, all contributing to effective tissue regeneration [24,25]. Additionally, Zn^2+^ exhibits potent antimicrobial properties, reducing bacterial adhesion and biofilm formation, major concerns in chronic wounds [26,27]. Despite these well-documented benefits, the integration of Zn^2+^ into alginate-based dressings remains underexplored, particularly in its ability to directly cross-link with alginate rather than being incorporated as ZnO nanoparticles [28].

Despite the advantages of traditional wound dressings, many are limited by insufficient bioactivity and poor infection control [29]. The surface modification of wound dressings represents a significant advancement in wound care, providing enhanced mechanical properties, controlled drug release, and superior interactions with biological tissues [30,31,32,33]. Integrating Zn^2+^ into alginate matrices offers a unique opportunity to leverage zinc’s wound-healing benefits while maintaining alginate hydrogels’ desirable properties [20]. These Zn^2+^-functionalized wound dressings exhibit prolonged antimicrobial effects, improved cellular adhesion, and faster tissue regeneration than conventional hydrogel dressings [34,35]. The ability to fine-tune the properties of these materials at the nanoscale level makes them highly adaptable for treating various wound types, from superficial injuries to deep chronic wounds requiring extended care [36,37,38].

Even though numerous studies explored alginate-based wound dressings, the direct comparison between zinc-crosslinked alginate (AlgZn) and sodium alginate (AlgNa) coatings on commercial dressings remains underexplored—particularly in the context of biofilm suppression and in vivo burn healing.

In this regard, this study introduces a facile, two-step surface functionalization strategy to fabricate WD@AlgZn dressings using Zn^2+^ ions, and evaluates their performance relative to both AlgNa-coated and uncoated controls. Unlike many previous studies that rely on zinc oxide nanoparticles, we employed direct ionic cross-linking with Zn^2+^ to retain hydrogel integrity and ensure a sustained, cytocompatible antimicrobial effect. The novelty of our work lies in integrating Zn^2+^ directly into an alginate hydrogel network coated onto traditional dressings, offering a scalable and biocompatible approach to next-generation wound care. This research advances the state of the art by demonstrating the superior antibacterial activity of dressings developed through simple yet effective methods.

Thus, the main objective of this study is to develop and evaluate zinc alginate-coated traditional wound dressings (WD@AlgZn) and to evaluate their antimicrobial properties and wound-healing performance in direct comparison to sodium alginate (WD@AlgNa) and conventional controls.

## 2. Results and Discussion

### 2.1. Physicochemical Characterization

**Figure 1:**
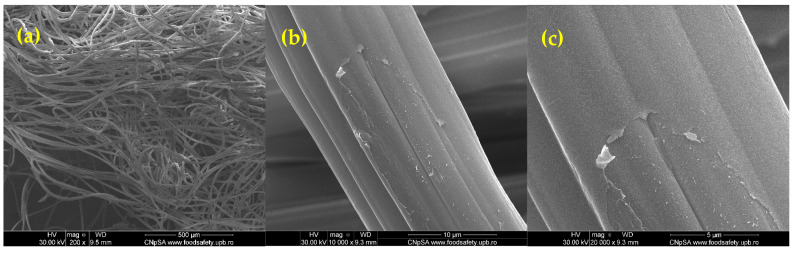
SEM analysis of WD@AlgZn: (**a**) magnification ×200, (**b**) magnification ×10,000, and (**c**) magnification ×20,000.

The SEM images (Figure 1) provide details about the structural morphology of the zinc alginate-coated wound dressings at different magnifications. A highly fibrous network is observed at a ×200 magnification (Figure 1a), characteristic of wound dressings designed for fluid absorption and oxygen permeability [39]. The fibers are randomly oriented, forming an interconnected structure that enhances wound healing by maintaining a moist environment [40]. At a ×5000 magnification (Figure 1b), individual fibers reveal a smooth surface with minor surface irregularities. Some regions show the presence of thin deposits of zinc alginate cross-linking residues. The fiber morphology suggests a well-distributed coating. Additionally, at a ×20,000 magnification (Figure 1c), small cracks and localized exfoliation can be noted, likely caused by the artificially induced mechanical stress.

**Figure 2:**
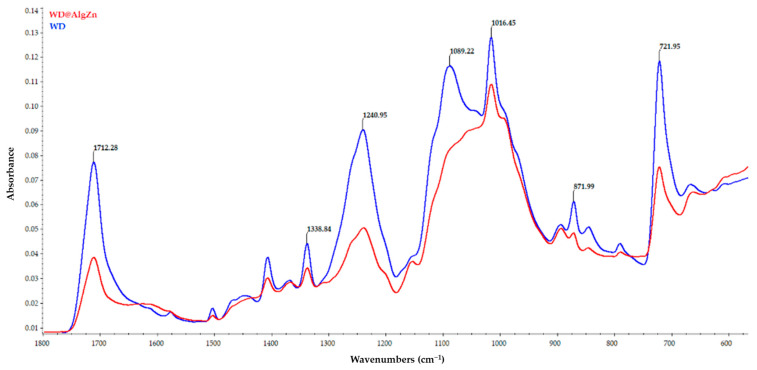
FT-IR spectra of WD and WD@AlgZn.

The FT-IR spectra (Figure 2) of the control wound dressing (WD) and the zinc alginate-coated wound dressing (WD@AlgZn) reveal structural and chemical modifications induced by the coating process. The WD (blue spectrum) exhibits characteristic absorption bands associated with its rayon–polyester composition, including a C=O stretching vibration at 1712 cm^−1^, which is linked to ester functional groups in polyester fibers. The 1240 cm^−1^ and 1016 cm^−1^ peaks correspond to C–O stretching vibrations, while 1338 cm^−1^ represents CH_2_ bending vibrations from the polymeric structure. The intense peak at 1089 cm^−1^, attributed to C–O–C stretching in the rayon (cellulose) and ester bonds in the polyester, is notably absent in the coated dressing. On the other hand, WD@AlgZn (red spectrum) shows significant spectral changes, confirming surface modification and interaction between the alginate and the dressing fibers. The carbonyl stretching peak at 1712 cm^−1^ decreases in intensity, indicating interactions between the alginate network and the polyester backbone. The peaks at 1240 cm^−1^ and 1016 cm^−1^, associated with C–O stretching, show shifts in position and intensity, suggesting Zn^2+^ coordination with the carboxylate (–COO^−^) groups of the alginate.

The disappearance of the 1089 cm^−1^ peak in the FT-IR spectrum of WD@AlgZn suggests a strong interaction between the zinc alginate coating and the rayon–polyester wound dressing matrix. This interaction is most likely due to the formation of a uniform alginate layer on the fiber surface, effectively modifying the surface chemistry and masking the underlying functional groups of the dressing. The alginate coating, cross-linked with Zn^2+^ ions, may create a continuous film over the fibers, preventing the infrared beam from detecting the characteristic C–O stretching vibration of cellulose (rayon) and ester groups (polyester). Zinc ions further enhance these interactions by coordinating with oxygen-containing functional groups in the dressing material, leading to molecular rearrangement and the suppression of specific vibrational modes.

Other possible explanations include the modification of glycosidic (C–O–C) and ester (C=O) bonds in the rayon–polyester structure due to interactions with the alginate hydrogel network, as well as cross-linking effects between the Zn^2+^ and the alginate carboxylate (–COO^−^) groups, which could alter the molecular environment of the wound dressing. These findings confirm the successful incorporation of zinc alginate into the dressing, highlighting its potential for enhanced mechanical stability, controlled bioactivity, and improved antimicrobial properties.

### 2.2. Antimicrobial Activity

**Figure 3:**
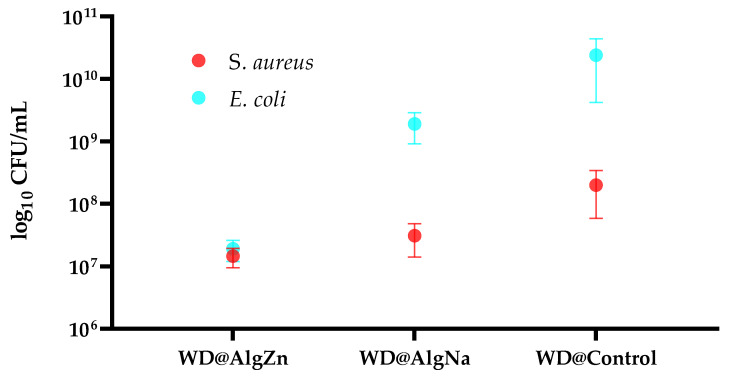
The evaluation of microbial adherence after 24 h of incubation in the presence and absence of dressings for the *E. coli* strain and *S. aureus* strain.

Figure 3 illustrates the antibacterial effects of different wound dressings by comparing the bacterial growth (log_10_ CFU/mL) of *Staphylococcus aureus* and *Escherichia coli* in the presence of WD@AlgZn, WD@AlgNa, and WD@Control. The results indicate that WD@AlgZn exhibits the lowest bacterial count for both strains, suggesting that incorporating Zn^2+^ significantly enhances antimicrobial properties. Zinc is known to interfere with bacterial metabolism, disrupt membrane integrity, and generate oxidative stress, ultimately inhibiting bacterial proliferation. The observed reduction in colony-forming units highlights the effectiveness of Zn^2+^ as an antimicrobial agent, particularly in controlling bacterial adhesion and biofilm formation, which are important factors in wound infection management.

In contrast, WD@AlgNa shows moderate bacterial inhibition, with bacterial counts higher than those observed for WD@AlgZn but still lower than the control. This indicates that sodium alginate alone provides some benefits due to its exudate-absorbing properties and ability to maintain a moist but controlled wound environment—however, the absence of Zn^2+^ results in reduced efficacy against bacterial colonization. The error bars have similar values in some experiments for WD@AlgNa, suggesting variability in bacterial growth and indicating that its effect may not be significantly different from the control in certain cases. WD@Control lacks any antimicrobial component, so it exhibits the highest bacterial load, particularly for *E. coli*, reaching nearly 10^10^ CFU/mL.

### 2.3. In Vivo Evaluation of Wound Healing in a Wistar Rat Burn Mode

#### Macroscopic Evaluation of Burn Wound Healing Following Topical Treatment

After one week of treatment, burn wounds treated with the WD@AlgZn exhibited a significantly improved healing progression compared to the three reference groups—the spontaneous healing control (REF) group, the 1% silver sulfadiazine (SDA) cream group, and the control dressing (WD_Control) group. Wound contraction and re-epithelialization occurred uniformly across the entire burned area, progressing from the periphery toward the center. In some cases, by day 21, the scab remained attached only by a small pedicle. Healing resulted in flat scars with minimal retraction, reaching near completion after 21 days of treatment. The wound-healing process in the WD@AlgZn group progressed at least 25% faster than in the REF, SDA, and WD_Control groups. In the REF and SDA groups, wound healing was delayed, with incomplete epithelialization still observed at 21 days. The WD_Control group exhibited a more favorable healing response compared to the REF group. Regarding initiating the healing process, wound appearance, and overall wound closure rate, the WD_Control group demonstrated an intermediate healing response between the REF and SDA groups (Table 1).

### 2.4. Microscopic Evaluation of Burn Wound Healing

**Figure 4:**
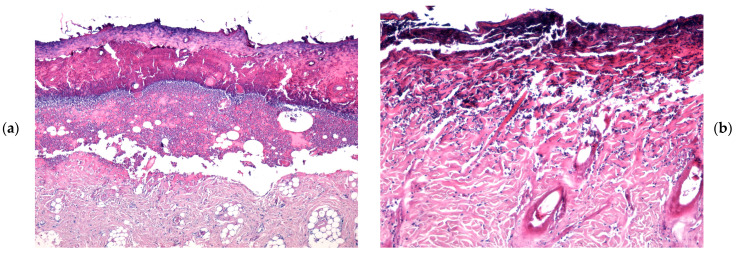

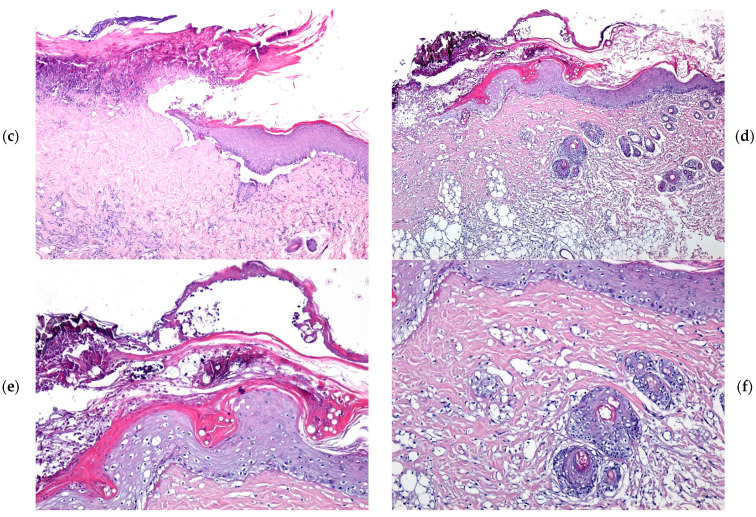
The microscopic evolution of skin burns after 7 days in the REF (**a**), SDA (**b**), WD_Control (**c**), and WD@AlgZn nanodressing (**d**–**f**) groups. Hematoxylin and eosin (H&E) staining: (**a**,**c**,**d**) ×40; (**b**,**e**,**f**) ×100.

At 7 days following burn induction, histological analysis revealed a thick coagulative necrosis zone at the wound surface, primarily composed of deformed collagen fibers exhibiting variable staining intensity. This necrotic layer was distinctly separated from the viable tissue by an inflammatory infiltrate, consisting predominantly of a dense band of polymorphonuclear neutrophils (PMNs). Additionally, remnants of pilosebaceous follicles showing marked degeneration were observed. The inflammatory response was notably reduced in wounds treated with the WD@AlgZn nanoparticle dressing, and post-burn edema was considerably less pronounced. In contrast, in the REF (spontaneous healing), SDA, and WD_Control groups, microscopic lesions were characterized by edematous vacuoles positioned between the necrotic zone and the muscle layer. Moreover, the inflammatory response in these groups was more extensive, in terms of the affected area and the number of PMN cells, compared to the WD@AlgZn-treated wounds (Figure 4a–c).

**Figure 5:**
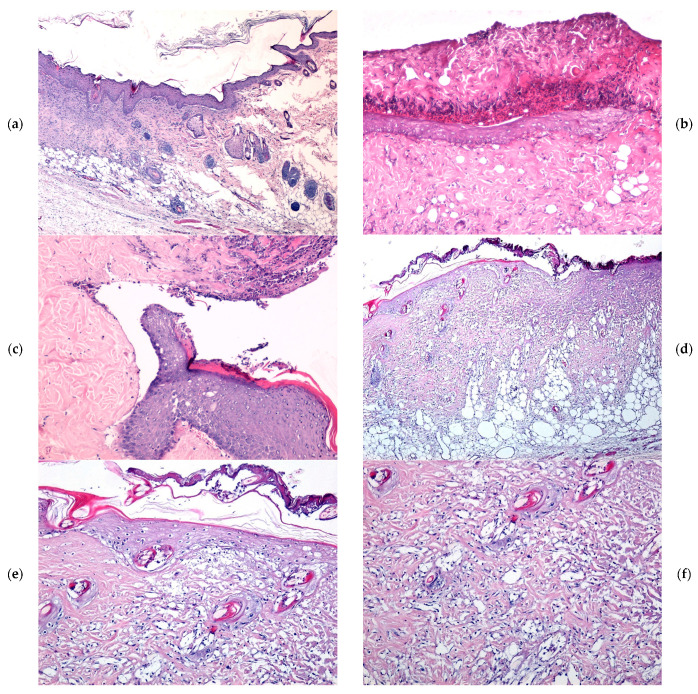
The microscopic evolution of skin burns after 14 days in the REF (**a**), SDA (**b**), WD_Control (**c**), and WD@AlgZn nanodressing (**d**–**f**) groups. H&E staining: (**a**,**d**) ×40; (**b**,**e**,**f**) ×100; (**c**) ×200.

By day 14, the wounds treated with the WD@AlgZn dressing exhibited a thinned coagulative necrosis layer, with an increased presence of immune system cells within the necrotic tissue and the underlying viable connective tissue. The boundary between the necrotic and viable tissue was marked by a fine band of PMNs and lymphocytes. Additionally, a dense vascular network indicative of active angiogenesis was observed within the deeper layers of the wound. In contrast, in the REF, SDA, and WD_Control groups, the coagulative necrosis layer remained relatively thick, with persistent edematous vacuoles observed within the necrotic region and the adjacent connective tissue. The separation between the necrotic zone and viable connective tissue was more pronounced, delineated by a thicker band of PMNs and lymphocytes. The inflammatory infiltrate was more extensive and widespread, and although neovascularization was present, it was less developed than in the WD@AlgZn-treated wounds (Figure 5a–c).

**Figure 6:**
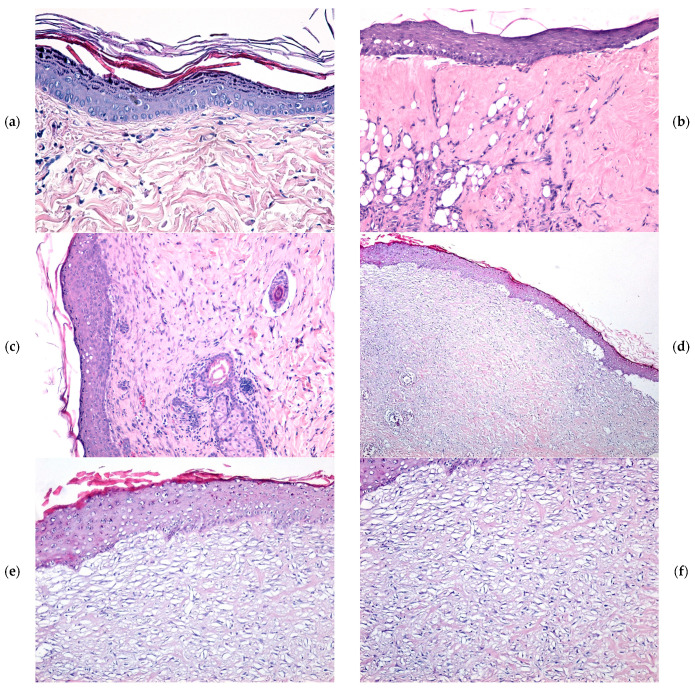
The microscopic evolution of skin burns after 21 days in the REF (**a**), SDA (**b**), WD_Control (**c**), and WD@AlgZn nanodressing (**d**–**f**) groups. H&E staining: (**d**) ×40; (**b**,**c**,**e**,**f**) ×100; (**a**) ×200.

At 21 days post injury, the granulation tissue in the WD@AlgZn-treated wounds exhibited significant remodeling. The inflammatory infiltrate was notably reduced, and neovascularization progressed towards maturation, characterized by the presence of well-formed blood vessels. Furthermore, the re-epithelialization process was well-defined, suggesting an advanced stage of tissue regeneration. In contrast, the REF, SDA, and WD_Control groups still exhibited persistent coagulative necrosis at the wound surface, with an underlying dense inflammatory infiltrate. The granulation tissue in these groups retained characteristics of immature tissue with a less advanced re-epithelialization process. Epithelial regeneration, although present, was incomplete and structurally deficient, characterized by a thin, discontinuous epithelial layer of variable thickness, indicating delayed wound closure compared to the WD@AlgZn-treated wounds (Figure 6a–c).

Chronic wounds and burn injuries present a significant challenge in clinical practice, often leading to prolonged healing times and an increased risk of infection [41,42]. While effective in basic wound coverage, traditional wound dressings often lack the bioactivity needed to actively promote tissue regeneration and antimicrobial defense [43]. Alginate-based wound dressings have gained attention due to their ability to maintain a moist healing environment and facilitate cell migration [44]. In particular, sodium alginate cross-linked with zinc ions (Zn^2+^) offers promising advantages, including enhanced mechanical stability, improved antimicrobial efficacy, and the stimulation of cellular proliferation [45,46,47].

The antimicrobial activity of zinc alginate-coated wound dressings was confirmed through in vitro studies, where significant bacterial inhibition was observed against *Staphylococcus aureus* and *Escherichia coli*. *S. aureus* is a Gram-positive bacterium with a thick peptidoglycan layer but no outer membrane, making it more susceptible to metal ion-induced oxidative stress and membrane damage. *E. coli*, a Gram-negative bacterium, has an outer lipopolysaccharide-rich membrane that acts as a barrier to certain antimicrobial agents. However, Zn^2+^ ions have been shown to disrupt this outer membrane by chelating negatively charged membrane components, increasing permeability, and enabling deeper ion penetration [48]. This explains the strong antimicrobial activity observed for WD@AlgZn against both bacterial strains, though the effects were more pronounced for *E. coli*, likely due to its greater initial biofilm formation potential.

Regarding the antibacterial mechanisms involved, zinc ions released from the dressings interfere with bacterial membrane permeability, disrupt enzymatic systems through protein denaturation, affect microbial metabolism, and generate reactive oxygen species (ROS), ultimately leading to bacterial cell death [48,49,50,51,52,53]. In the developed dressings, Zn^2+^ is ionically cross-linked within the alginate matrix, enabling a sustained release profile that ensures prolonged antimicrobial action. This prolonged activity helps prevent biofilm formation, which is a major challenge in chronic wound infections [54,55,56]. The antibacterial properties of zinc also reduce the need for conventional topical antibiotics, such as mupirocin, neomycin, bacitracin, and silver sulfadiazine, which helps mitigate the rise in antibiotic-resistant bacterial strains in wound care management [57,58,59,60,61]. In contrast, WD@AlgNa lacks intrinsic antibacterial components; its mild inhibition likely stems from moisture control and biofilm suppression due to the alginate’s hydrophilic properties [62,63,64], but it is far less effective compared to Zn^2+^-containing dressings.

The controlled release of Zn^2+^ ions is also important in ensuring cellular compatibility, as excessive concentrations may induce cytotoxic effects, such as oxidative stress and mitochondrial dysfunction, if systemic absorption occurs in significant amounts [65,66]. Nonetheless, in the case of the WD@AlgZn dressing, the ionic cross-linking within the alginate matrix ensures that zinc is primarily released in a localized, sustained manner at the wound site, which minimizes systemic exposure. Moreover, studies indicate that optimal Zn^2+^ release enhances fibroblast migration, extracellular matrix deposition, and angiogenesis, all contributing to accelerated wound healing [24]. Additionally, Zn^2+^ modulates inflammatory responses by regulating cytokine production and reducing excessive inflammation, thereby creating a more favorable environment for tissue regeneration [25,67]. Additionally, clinical trials involving zinc-based wound care products (e.g., zinc oxide or zinc sulfate dressings) have reported minimal adverse effects, typically limited to mild local irritation in sensitive individuals [68]. Thus, the use of Zn^2+^ in WD@AlgZn is not only biocompatible but also unlikely to pose systemic risks when applied to localized injuries under controlled clinical conditions.

The in vivo wound-healing assessment further corroborated these findings, as wounds treated with WD@AlgZn dressings exhibited enhanced re-epithelialization, faster granulation tissue formation, and improved collagen deposition compared to untreated wounds. Promoting angiogenesis is particularly significant, as zinc has been shown to stimulate vascular endothelial growth factor expression, facilitating new blood vessel formation and thereby improving nutrient and oxygen supply to the wound site [69,70]. These results highlight the ability of WD@AlgZn dressings to accelerate wound closure and reduce healing time, which is necessary in clinical applications for burn injuries and chronic wounds [53,71].

A key advantage of WD@AlgZn wound dressings is their ability to balance inflammatory responses. Excessive inflammation considerably impacts wound healing, leading to prolonged tissue damage and fibrosis [72]. Zn^2+^ plays a dual role by exerting antimicrobial effects while simultaneously downregulating inflammatory cytokines such as TNF-α, IL-6, and IL-1β, leading to controlled immune responses and enhanced healing outcomes [52,73]. Additionally, zinc has been implicated in matrix metalloproteinase regulation, further contributing to controlled extracellular matrix remodeling and reduced scarring [74,75,76].

Despite these advantages, challenges remain regarding the long-term stability and controlled release of Zn^2+^ from the alginate matrix. Overly rapid ion release can lead to local cytotoxic effects, whereas slow release may reduce therapeutic efficacy [77]. Although formal stability testing was not within the scope of this study, the structural integrity and antimicrobial functionality of the WD@AlgZn and WD@AlgNa dressings are expected to remain stable under typical storage conditions (ambient temperature, dry, and protected from light) for at least several weeks. In particular, alginate-based hydrogels cross-linked with divalent cations, such as Zn^2+^, are known to exhibit enhanced matrix rigidity and reduced hydrolytic degradation [78,79], thus strengthening the gel network, slowing down alginate dissolution in aqueous environments, and improving shelf life stability and controlled Zn^2+^ release during application. Nevertheless, we acknowledge that stability evaluation represents a limitation of this work and that further quantitative studies on long-term stability, moisture retention, and Zn^2+^ release kinetics under storage and application conditions would provide more insight and will be the subject of future work.

In summary, WD@AlgZn exhibits strong antimicrobial properties and significant wound-healing potential. These properties make it a promising candidate for next-generation wound care applications, particularly in managing chronic wounds, burns, and diabetic ulcers.

## 3. Conclusions

This study highlights the potential of zinc alginate-coated traditional wound dressings as an effective and biocompatible solution for wound management. The integration of zinc ions into the alginate matrix significantly enhances the antimicrobial properties while maintaining the inherent biocompatibility of the alginate structure. The in vivo assessments confirmed that the zinc alginate dressings provide superior healing outcomes compared to conventional dressings, reducing inflammation and accelerating tissue regeneration.

## 4. Materials and Methods

### 4.1. Materials

All chemical reagents used in this study were of analytical grade and were procured from Sigma-Aldrich Merck (Darmstadt, Germany). The sodium alginate and zinc nitrate (Zn(NO_3_)_2_) were used as received, without additional purification, to ensure consistency and reproducibility in all experimental procedures.

### 4.2. Preparation of Zinc Alginate-Coated Wound Dressings

Wound dressing samples (rayon–polyester-based) were initially coated with sodium alginate, followed by cross-linking with copper ions in a subsequent step. For this study, wound dressings were cut into standardized dimensions of 1 cm × 1 cm. To fabricate zinc alginate ((Alg)_2_Zn) dressings, the process was conducted in two distinct stages. In the first stage, sodium alginate (AlgNa) was accurately weighed and dissolved in distilled water under continuous magnetic stirring to ensure complete solubilization (1% *w*/*v*). The wound dressing samples were immersed in the prepared AlgNa solution and then removed, resulting in AlgNa-coated dressings. In the second stage, the AlgNa-coated dressings were further immersed in a Zn^2+^ solution prepared by dissolving Zn(NO_3_)_2_ in distilled water under magnetic stirring (10% *w*/*v*). This process facilitated the ionic exchange necessary for zinc alginate hydrogel formation. The immersion time was fixed at 15 min under continuous gentle stirring to ensure cross-linking and stable hydrogel formation. This time frame was selected based on preliminary optimization to ensure consistent gel formation without over-saturation. After immersion, the samples were thoroughly rinsed with distilled water to remove excess reagents and dried at room temperature for 24 h. The alginate-coated samples were weighed after cross-linking and complete drying, and the alginate mass was determined to be 1.0 ± 0.01 mg per cm^2^ of wound dressing.

### 4.3. Physicochemical Characterization Methods

A scanning electron microscope purchased from FEI Company (Hillsboro, OR, USA) was utilized to study the structural features and scale of the nanostructured dressing samples. The micrographs were taken by detecting secondary electron signals at an accelerating voltage of 30 keV.

A Nicolet iN10 MX FT-IR spectrometer (ThermoFisher Scientific, Waltham, MA, USA) was used to record infrared spectra over a 4000–300 cm^−1^ wavenumber range in order to investigate the integrity of the functional groups characteristic to developed materials. Measurements were carried out in reflection mode with a resolution of 4 cm^−1^. For each sample, 32 scans were collected, combined, and analyzed using OmnicPicta 8.2 software (Thermo Scientific, Waltham, MA, USA).

### 4.4. In Vivo Experimental Model

All experimental procedures complied with European Council Directive 86/609 (24 November 1986), the European Convention for the Protection of Vertebrate Animals Used for Experimental and Other Scientific Purposes (2 December 2005), and Romanian Law No. 43 (11 April 2014) concerning the protection of animals used in scientific research. The study received ethical approval from the Bioethics Committee of the University of Medicine and Pharmacy of Craiova, Romania (Approval Report No. 118/27 May 2015).

### 4.5. Animal Model and Experimental Design

The study involved four groups of male Wistar rats, each consisting of 10 animals, aged eight weeks, with an average body weight of 350 ± 10 g. Throughout the experimental period, the animals were housed in the Animal Facility of the University of Medicine and Pharmacy of Craiova under standard conditions of temperature, humidity, and light cycles, with free access to food and water (ad libitum). General anesthesia was administered via the intramuscular injection of a ketamine hydrochloride and xylazine hydrochloride combination (85 mg/kg body weight, Ketamidor^®^ 100 mg/mL; Richter Pharma AG, Wels, Austria, and 6 mg/kg body weight, Xylazin Bio^®^ 2%; Bioveta, Nitra, Slovakia). Following anesthesia, the dorsal fur was shaved, and full-thickness third-degree burns were induced over an area of approximately 1.5 cm^2^ using a 350 g conical metal device with a 1 cm diameter. The device was preheated to 100 °C and applied to the skin for 5 s. Functionalized nanostructured dressings were applied immediately after burn induction and were changed daily for three weeks. To evaluate wound healing, four experimental groups were established. In the reference group (REF), the burns were left to heal spontaneously, covered only with a standard gauze dressing moistened with physiological saline. In the second group (SDA), the burns were treated with a 1% silver sulfadiazine cream (Dermazin^®^, Sandoz, Basel, Switzerland). In the control group (WD_Control), the burns were covered with a dressing containing a hydrogel with sodium alginate alone. In the fourth group (WD@AlgZn), a dressing substrate with immobilized nanostructures was applied to the burn wounds under the same experimental conditions [80].

### 4.6. Wound-Healing Monitoring

The evolution of the burn wounds was monitored daily over three weeks through the macroscopic evaluation of inflammation indicators, including edema, erythema, and re-epithelialization. Initially, the burns presented with epithelial necrosis, the destruction of the underlying connective tissue extending to the muscle layer, the perilesional edema of approximately 4 mm, and intense erythema [80].

The wound-healing rate (%) was calculated by the difference between the area of the original wound and the area of the remaining wound divided by the area of the original wound (Healing rate (%) = (area of original wound size − area of the remaining wound)/area of the original wound size) [41].

### 4.7. Histological Analysis

At 7, 14, and 21 days after burn induction, the animals were anesthetized for tissue collection. The burned tissue was promptly rinsed with phosphate-buffered saline (PBS) to remove residual blood, then fixed in 10% neutral buffered formalin at room temperature for 72 h. The samples were subsequently embedded in paraffin for microscopic morphological analysis [80].

For histological analysis, serial sections of a thickness of 4 µm were prepared using a MICROM HM355s rotary microtome (MICROM International GmbH, Walldorf, Germany) equipped with a section transfer system (STS, microM). The sections were mounted on poly-L-lysine-coated glass slides (Sigma-Aldrich, Munich, Germany). Following hematoxylin and eosin (H&E) staining, the slides were examined and imaged using a Nikon Eclipse 55i optical microscope equipped with a high-definition Nikon DS-Fi1 CCD camera (Nikon Instruments, Apidrag, Bucharest, Romania). Image acquisition, storage, and processing were carried out using Image-Pro Plus 7 AMS software (Media Cybernetics Inc., Marlow, UK) [80].

### 4.8. Statistical Analysis

For statistical analysis, Student’s *t*-test was applied, and the experimental values are expressed as mean ± standard deviation (SD). For graph plotting, Origin 8 (OriginLab, Northampton, MA, USA) software was used. *p* < 0.05 differences were considered statistically significant. Each experiment was performed in triplicate.

### 4.9. Antimicrobial Activity

The bacterial strains used in this study—*Staphylococcus aureus* ATCC 25923 and *Escherichia coli* ATCC 25922—were sourced from the strain collection of the Microbiology Laboratory, Faculty of Biology, University of Bucharest. To assess the impact of the coated surfaces on biofilm formation, the materials were cut into standardized 1 cm × 1 cm samples and sterilized by UV exposure for 20 min on each side. A sterile piece of each material was placed in a separate well of a sterile six-well plate. Subsequently, 2 mL of liquid medium (simple broth) was added to each well, followed by the inoculation of 50 μL of a microbial suspension standardized to 0.5 McFarland density. The plates were then incubated at 37 °C for 24 h.

After incubation, the materials were rinsed with phosphate-buffered saline (PBS), and the culture medium was replaced to support continued biofilm development. The plates were further incubated for 24, 48, or 72 h, according to the designated experimental time points. At the end of each incubation period, the samples were washed with PBS and transferred to sterile tubes containing 1 mL of PBS. To detach the biofilm cells from the material surfaces, the tubes were vortexed vigorously for 30 s. The resulting cell suspensions were serially diluted and plated on solid culture medium to quantify colony-forming units (CFUs). This method enabled the assessment of biofilm formation on the tested surfaces across different time intervals.

## Figures and Tables

**Table 1:** Macroscopic evolution of burn wounds following dressing application.

**Day**	**REF**	**SDA**	**WD_Control**	**WD@AlgZn**
	**Burn Wound Area (mean ± SD) (cm^2^)/Healing Rate (%)**			
7	1.28 ± 0.15/15.33 **	1.18 ± 0.12/25.48 **	1.34 ± 0.15/19.75 **	1.32 ± 0.13/38.25 **
14	0.95 ± 0.14/28.66 **	0.68 ± 0.06/41.25 **	0.84 ± 0.10/36.33 **	0.56 ± 0.10/71.33 **
21	0.56 ± 0.08/37.25 *	0.29 ± 0.02/52.32 *	0.38 ± 0.02/46.58 *	0.18 ± 0.02/90.75 *

REF: spontaneous healing control group; SDA: group treated with 1% silver sulfadiazine cream; WD_Control: control dressing group (wound-dressing control); WD@AlgZn: group treated with WD@AlgZn dressing; SD: standard deviation; * *p* < 0.01; ** p < 0.05.

## Data Availability

The original contributions presented in this study are included in the article. Further inquiries can be directed to the corresponding author.
